# The River's Tale

**Published:** 1919-08-09

**Authors:** Rudyard Kipling


### "The River's Tale."

**By RUDYARD KIPLING.**

*Twenty bridges from Tower to Kew*
*Wanted to know what the River knew,*
*For they were young and the Thames was old,*
*And this is the tale that the River told:*

I walk my beat before London town,
Five hours up and seven down.
Up I go and I end my run
At Tide-end-town, which is Teddington.
Down I come with the mud in my hands
And plaster it over the Maplin Sands.
But I'd have you know that these waters of mine
Were once a branch of the River Rhine,
When hundreds of miles to the East I went
And England was joined to the Continent.

I remember the bat-winged lizard-birds,
The Age of Ice and the mammoth herds,
And the giant tigers that stalked them down
Through Regent's Park into Camden Town.
And I remember like yesterday
The earliest Cockney who came my way,
When he pushed through the forest that lined the Strand,
With paint on his face and a club in his hand.
He was death to feather and fin and fur,
He trapped my beavers at Westminster,
He netted my salmon, he hunted my deer,
He killed my herons off Lambeth Pier;
He fought his neighbour with axes and swords,
Flint or bronze, at my upper fords,
While down at Greenwich for slaves and tin
The tall Phœnician ships stole in,
And North Sea war-boats, painted and gay,
Flashed like dragon-flies Erith way;
And Norseman and Negro and Gaul and Greek
Drank with the Britons in Barking Creek,
And life was gay, and the world was new,
And I was a mile across at Kew!
But the Roman came with a heavy hand,
And bridged and roaded and ruled the land,
And the Romans left and the Danes blew in—
And that's where your history books begin!

